# Lower short- and long-term mortality associated with overweight and obesity in a large cohort study of adult intensive care unit patients

**DOI:** 10.1186/cc11903

**Published:** 2012-12-18

**Authors:** Swapna Abhyankar, Kira Leishear, Fiona M Callaghan, Dina Demner-Fushman, Clement J McDonald

**Affiliations:** 1 Lister Hill National Center for Biomedical Communications, National Library of Medicine National Institutes of Health, 8600 Rockville Pike, Building 38A/7N707, Bethesda, MD 20894, USA

## Abstract

**Introduction:**

Two thirds of United States adults are overweight or obese, which puts them at higher risk of developing chronic diseases and of death compared with normal-weight individuals. However, recent studies have found that overweight and obesity by themselves may be protective in some contexts, such as hospitalization in an intensive care unit (ICU). Our objective was to determine the relation between body mass index (BMI) and mortality at 30 days and 1 year after ICU admission.

**Methods:**

We performed a cohort analysis of 16,812 adult patients from MIMIC-II, a large database of ICU patients at a tertiary care hospital in Boston, Massachusetts. The data were originally collected during the course of clinical care, and we subsequently extracted our dataset independent of the study outcome.

**Results:**

Compared with normal-weight patients, obese patients had 26% and 43% lower mortality risk at 30 days and 1 year after ICU admission, respectively (odds ratio (OR), 0.74; 95% confidence interval (CI), 0.64 to 0.86) and 0.57 (95% CI, 0.49 to 0.67)); overweight patients had nearly 20% and 30% lower mortality risk (OR, 0.81; 95% CI, 0.70 to 0.93) and OR, 0.68 (95% CI, 0.59 to 0.79)). Severely obese patients (BMI ≥ 40 kg/m^2^) did not have a significant survival advantage at 30 days (OR, 0.94; 95% CI, 0.74 to 1.20), but did have 30% lower mortality risk at 1 year (OR, 0.70 (95% CI, 0.54 to 0.90)). No significant difference in admission acuity or ICU and hospital length of stay was found across BMI categories.

**Conclusion:**

Our study supports the hypothesis that patients who are overweight or obese have improved survival both 30 days and 1 year after ICU admission.

## Introduction

Two thirds of the U.S. adult population is obese (body mass index (BMI), ≥ 30 kg/m^2^) or overweight (BMI, 25 to < 30 kg/m^2^) [1]. Obesity is associated with a higher risk of developing chronic diseases, including diabetes, hypertension, osteoarthritis, and coronary artery disease (CAD) compared with normal weight (BMI, 18.5 to < 25 kg/m^2^), and epidemiologic studies have found that obese adults have significantly higher mortality than do those with a BMI between 20 and 25 kg/m^2 ^[2]. One national study found that both obesity and underweight were associated with significantly higher all-cause mortality [3]. However, obesity might be associated with increased mortality only in patients who also have a comorbid condition: a follow-up study by the same group found that only obese patients with cardiovascular disease (CVD), obesity-related cancer, diabetes, or kidney disease had higher mortality [4]. In a separate long-term observational study on the natural progression of cardiovascular disease, researchers found that obesity did not increase the risk of organ failure or in-hospital mortality for those that developed organ failure, and that diabetes was a significant predictor of both, regardless of weight status [5].

Studies looking directly at the effect of obesity on mortality after admission to the intensive care unit (ICU) had mixed results. Some investigators found that obese individuals had higher mortality during critical illness [6-9], whereas others reported that obesity either had no effect [10-14] or was protective compared with normal weight [15-18]. The studies that found obesity protective were either small (< 1,000 patients) [16], focused on a subset of patients in the ICU (for example, surgical ICU patients [17], patients with acute lung injury who were mechanically ventilated [18]), or excluded nearly half of the potentially large study population because of missing data [15]. Two large meta-analyses also had mixed results. Oliveros and Villamor [19] reviewed 23 studies and found that overweight and obesity, but not severe obesity (BMI ≥ 40 kg/m^2^), were linked to lower ICU or hospital mortality (they did not separate the studies that reported ICU mortality from those that reported hospital mortality) [19], whereas Hogue and colleagues [20] reviewed 22 studies (most of which were included in the meta-analysis of Oliveros and Villamor) and found that overweight and obesity had no effect on ICU mortality but were associated with lower hospital mortality.

Most of the previous studies looked at ICU and/or in-hospital mortality, which can be misleading because of hospital transfer and discharge practices geared toward improving performance measures (for example, in-hospital mortality rate) [21]. Few studies have addressed the effect of BMI on mortality associated with critical illness at or beyond 30 days after the hospitalization. Also, many did not use the standard World Health Organization (WHO) BMI categories [22] or compared outcomes of obese patients with those of a reference group that included not only normal-weight patients but also underweight and/or overweight patients [7-9,11,12,14], which may have biased the results, as underweight patients tend to have worse survival than average. Last, most studies adjusted for only a limited number of obesity-related conditions and other potential confounders.

We used the Multiparameter Intelligent Monitoring in Intensive Care (MIMIC-II) database [23] to examine the relation between BMI and mortality 30 days and 1 year after ICU admission. MIMIC-II contains clinical ICU data on > 25,000 patients as well as death data for 1 year after hospital discharge. The size, comprehensive content, and long-term mortality information contained in this database may provide the means to clarify the direction of the association between obesity, obesity-related conditions, and mortality related to ICU hospitalization.

## Methods and materials

The MIMIC-II database, maintained by the Laboratory for Computational Physiology at the Massachusetts Institute of Technology (MIT), contains data on patients hospitalized in an ICU at Beth Israel Deaconess Medical Center from 2001 to 2008. The database is freely available, in that any researcher who accepts the data-use agreement and has completed "protecting human subjects" training can apply for permission to access the data [24]. We did not need patient consent or ethics approval, as all of the data are deidentified. All of the authors completed the "protecting human subjects" training. We conducted the study under National Institutes of Health Institutional Review Board exemption number 4193.

The MIMIC-II data were deidentified according to the Health Insurance Portability and Accountability Act Privacy Rules [25]. The deidentification process included random date shifting that preserved the temporal relation with a given patient but not across patients. The database includes basic admission and demographic information as well as vital signs, laboratory and radiology results, medications, discharge diagnoses, nursing notes, physician discharge summaries, and dates of death. MIMIC-II contains patients from five ICU types: medical (MICU), surgical (SICU), cardiac (CCU), cardiac surgery recovery (CSRU), and neonatal (NICU). We used data from version 2.5 and 2.6 of MIMIC-II, as each version contained information not available in the other.

**Figure 1:**
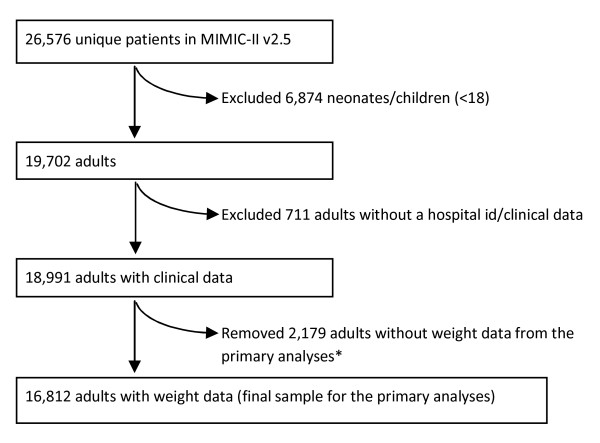
**Study cohort selection**. Cohort selection and criteria for exclusion. *The primary analyses did not include the patients without weight data; a subsequent sensitivity analysis included those patients based on imputed values for BMI.

MIMIC-II contains information on 26,576 unique patients (see Figure 1). The data were originally collected during the course of clinical care, and we subsequently extracted our dataset independent of the study outcome. We excluded the 6,874 nonadults (primarily neonates) from the analyses. We also excluded 711 patients who lacked the dummy identifiers needed to link to their clinical data. Of the remaining 18,991, we removed 2,179 (11%) patients who had no weight recorded, for a final total of 16,812 patients for our final analysis.

We used weight and height values recorded in the ICU, and, if needed, from echocardiogram reports and reverse-computed from a combination of body-surface-area and weight measurements. Of the 16,812 adult patients with weights, 4,223 (25%) were missing height values. We imputed height values for these patients by using the sample median height for their age and gender plus or minus a random value based on the sample standard deviation. BMI was calculated as (weight (in kilograms)/height (in meters)^2^). We grouped patients into the four major WHO BMI categories: < 18.5 kg/m^2 ^(underweight), 18.5 to < 25 kg/m^2 ^(normal weight), 25 to < 30 kg/m^2 ^(overweight), and ≥ 30 kg/m^2 ^(obese) [22]. We also performed a subgroup analysis on the WHO obese class III patients, defined as having a BMI ≥ 40 kg/m^2 ^or "severely obese," because they have an even higher risk of developing chronic conditions [2] and of all-cause mortality compared with obese patients with a BMI of 30 to < 40 kg/m^2 ^[26]. Often the severely obese are not studied as a group because of insufficient sample size, but the MIMIC-II database gave us the opportunity to study this group separately.

We used primarily ICD-9 discharge diagnoses to determine whether patients had obesity-related conditions including diabetes mellitus, CVD, obesity-related cancer, kidney disease, and osteoarthritis. We also used information about oral diabetes medications to augment the diabetes ICD-9 codes. We split CVD into four categories: CAD, hypertension, stroke, and "other CVD" (that is, CVD but not CAD, hypertension, or stroke) to isolate the effects of each. We used the National Cancer Institute Obesity and Cancer Fact Sheet [27] to define obesity-related cancers as colon, breast, esophageal, uterine, ovarian, kidney, and pancreatic cancer.

We calculated the Simplified Acute Physiology Score (SAPS) [28], a measure of patient acuity for the first 24 hours of the ICU admission, to assess whether patients in different BMI categories had different levels of disease severity when they were admitted to the ICU. The SAPS is based on the worst recorded value for 14 physiological and laboratory parameters, including age, certain vital signs, and laboratory results such as specific electrolytes, hematocrit, and white cell count, during the first 24 hours of the admission. We calculated the SAPS for each patient from the MIMIC-II data. Ninety-four percent of patients had data for all 14 SAPS components. Of the remaining 6%, 3% had a single component missing, and the remaining 3% had two or more components missing. For those that had one or more components missing, we added results for the missing components from 6 hours before ICU admission, when available, based on the assumption that if a patient had a laboratory test a few hours before ICU admission, the test typically would not be repeated until the next day. After addition of these values, 95% had complete SAPS data, 3% had one component missing, and only 2% had two or more components missing. For the final analysis, as specified by the SAPS algorithm, we assumed a normal score for the missing components.

Although SAPS is a good measure of patient acuity on admission to the ICU, it does not reflect a patient's baseline health status before events that led to hospitalization. SAPS II, the updated version of SAPS, includes a score to account for prior health status based on the presence or absence of the following chronic conditions: blood cancers (leukemia, lymphoma, multiple myeloma), metastatic cancer, and HIV [29]. We did not calculate the entire SAPS II score because we did not have enough data for the FiO_2 _and PaO_2 _measurements necessary to calculate SAPS II that were not in the original SAPS. However, we used ICD-9 codes to determine whether patients had one of the three chronic conditions specified by SAPS II to see if a difference in baseline health existed across the BMI categories.

### Statistical methods

Our statistical methods included the following steps, which are described in further detail later: (a) univariate analysis to describe the study variables by BMI category and to estimate crude mortality risk by BMI category before adjusting for any covariates; (b) multivariable logistic regression analyses to predict mortality risk by BMI category after adjusting for a large set of covariates; (c) sensitivity analyses to determine whether our approach and assumptions about missing data distorted our results; and (d) Cox proportional hazards models to confirm the findings of the multivariable logistic regression models.

#### Univariate analysis

We used univariate analysis to describe study variables by BMI category and to determine whether each variable was significantly different across BMI categories. We used the Jonckheere-Terpstra test for trend for categoric variables and the ANOVA test for trend for continuous variables. In addition, we used χ^2 ^tests or Fisher Exact tests for categoric variables and *t *tests for continuous variables to compare each of the underweight, overweight, and obese categories with the normal-weight group. We constructed two univariate logistic regression models, with BMI category as the predictor: one had 30-day mortality, and the other had 1-year mortality as the outcome.

#### Multivariable regression analysis

We chose the covariates described later, based on clinical knowledge as well as the previously published literature on obesity and critical illness. We built the multivariable models by adding groups of covariates that were likely to be potential confounders to the univariate model one at a time in the following order: demographics (age, sex, and race), obesity-related conditions (diabetes, CAD, stroke, hypertension, other CVD, obesity-related cancers, kidney disease, and osteoarthritis), type of admission, ICU unit, SAPS score, ICU interventions and complications (ventilation, transfusion, total parenteral nutrition (TPN), sepsis, and pulmonary embolism (PE)), smoking status, marital status, and health insurance. Variables were removed only if *P *> 0.10. Multicollinearity for independent variables was assessed by using the variance inflation factor (VIF); no VIF was > 2.

#### Sensitivity and Cox proportional hazards analyses

We performed several additional analyses to determine whether our approach and assumptions about missing data distorted our results. First, we dealt with any potential bias of using imputed heights for patients with missing height values by removing these patients and rerunning the final multivariable regression models without them.

Second, we imputed BMIs for the 2,179 patients without weight data whom we had excluded from the primary study population and reran the analysis. We assigned the normal BMI category to these patients because overall, they were a healthier group, and adding them to the normal BMI group was most likely to reverse our conclusions.

Third, we removed the underweight group and reanalyzed the final models to see whether the underweight (high-mortality) patients skewed the results and whether removing them would reduce the mortality advantage of obese and overweight patients compared with those with normal weight. In addition, we did a subgroup analysis of the severely obese patients as a separate group.

Fourth, we created a comprehensive CVD variable that combined CAD, hypertension, stroke, and other CVDs to determine whether collinearity (a statistical modeling issue in which high correlation among similar predictors tends to inflate the estimates of risk in the regression model) affected the results.

Fifth, we did separate analyses to see whether any of the obesity-related conditions, admission SAPS, or ICU interventions mediated the relation between BMI category and mortality risk. We added each potential mediator sequentially to the model to observe the attenuation effect on the odds ratios for each BMI category. We performed an additional analysis to see whether certain medications used to treat obesity-related conditions (β-blockers, statins, and insulin) were potential confounders in our analysis, based on a recent article by Iozzo and colleagues [30]. We did not include oral diabetes medications as a potential confounder because those medications, along with ICD-9 codes, were used by us to define patients with diabetes. Finally, we analyzed each ICU unit separately to see whether the association between BMI and mortality was the same across the unit types.

Finally, we repeated the analysis of the final logistic regression models by using Cox proportional hazards regression to see whether the results persisted with a change in the method of analysis.

We used the SAS v9.2 (SAS Institute Inc, Cary, NC, USA) FREQ, UNIVARIATE, TTEST, and GLM procedures for univariate analysis, the LOGISTIC procedure for the logistic regression analysis, and the PHREG procedure for the Cox models. We used R v2.11.1 (R Foundation for Statistical Computing, Vienna, Austria) to create Kaplan-Meier curves and calculate the number at risk.

## Results

Univariate analysis of demographic and clinical characteristics by BMI category is shown in Tables 1 and 2, respectively. More than 30% of patients were in each of the normal, overweight, and obese categories, and approximately 5% were underweight. Obese patients were younger and more likely to be married compared with the normal-weight group (*P *< 0.001 for each). The overweight and obese patients were more likely to have private insurance compared with normal-weight patients (*P *< 0.001).

**Table 1:** Univariate analysis of demographic characteristics by body mass index category.

	Total	Underweight (BMI < 18.5 kg/m^2^)	Normal (BMI 18.5 to < 25 kg/m^2^)	Overweight (BMI 25 to < 30 kg/m^2^)	Obese (BMI ≥ 30 kg/m^2^)	*P *value^a^
***n *(%)**	16,812	786 (4.7)	5,463 (32.5)	5,276 (31.4)	5,287 (31.4)	

**Age (years), *n *(%)**						< 0.001

< 45	2,522 (15.0)	118 (15.0)	905 (16.6)	757 (14.4)^b^	742 (14.0)^b^	< 0.001

45-65	5,593 (33.3)	219 (27.9)	1,467 (26.9)	1,677 (31.8)^b^	2,230 (42.2)^b^	< 0.001

65-80	5,393 (32.1)	215 (27.4)	1,673 (30.6)	1,814 (34.4)^b^	1,691 (32.0)	0.01

80+	3,304 (19.7)	234 (29.8)^b^	1,418 (26.0)	1,028 (19.5)^b^	624 (11.8)^b^	< 0.001

**Age (years), median (Q1-Q3)**	65.9 (52.3, 77.8)	70.6 (53.0, 81.8)	69.4 (51.7, 80.4)	67.2 (53.6, 77.8)	62.3 (51.7, 73.2)	< 0.001

**Female, *n *(%)**	7,170 (42.7)	443 (56.4) ^b^	2,469 (45.2)	1,901 (36.0)^b^	2,357 (44.6)	< 0.001

Race, *n *(%)						< 0.001

White	11,390 (82.9)	534 (77.3)^b^	3,667 (81.5)	3,606 (84.3)^b^	3,583 (83.8)^b^	< 0.001

Black	1,179 (8.6)	71 (10.3)^b^	355 (7.9)	333 (7.8)	420 (9.8)^b^	0.02

Hispanic or Latino	430 (3.1)	16 (2.3)	148 (3.3)	144 (3.4)	122 (2.9)	0.58

Asian	322 (2.3)	47 (6.8)^b^	173 (3.8)	72 (1.7)^a^	30 (0.7)^b^	< 0.001

Other	424 (3.1)	23 (3.3)	157 (3.5)	121 (2.8)	123 (2.9)	0.09

Marital status, *n *(%)						< 0.001

Married	8,317 (53.7)	271 (38.6)^b^	2,524 (50.6)	2,819 (57.8)^b^	2,703 (55.0)^b^	< 0.001

Single/Divorced/Separated	4,690 (30.3)	278 (39.5)^b^	1,545 (31.0)	1,359 (27.9)^b^	1,508 (30.7)	0.02

Widowed	2,479 (16.0)	154 (21.9)^b^	923 (18.5)	699 (14.3)^b^	703 (14.3)^b^	< 0.001

Insurance, *n *(%)						< 0.001

Medicare/Medicaid^c^	8,806 (52.4)	501 (63.7)^b^	3,091 (56.6)	2,658 (50.4)^b^	2,556 (48.3)^b^	< 0.001

Private	6,724 (40.0)	231 (29.4)^b^	1,877 (34.4)	2,221 (42.1)^b^	2,395 (45.3)^b^	< 0.001

Other	1,282 (7.6)	54 (6.9)^b^	495 (9.1)	397 (7.5)^b^	336 (6.4)^b^	< 0.001

BMI, body mass index. ^a^*P *value for association or trend across BMI categories; ^b^*P *< 0.05 compared with normal weight. ^c^Medicare/Medicaid are state or federal programs in the United States that provide insurance for the elderly or patients with low income or significant chronic health conditions.

**Table 2:** Univariate analysis of hospitalization characteristics, chronic conditions, and mortality by body mass index category.

	Total	Underweight (BMI < 18.5 kg/m^2^)	Normal weight (BMI 18.5 to < 25 kg/m^2^)	Overweight (BMI 25 to < 30 kg/m^2^)	Obese (BMI ≥ 30 kg/m^2^)	*P *value^a^
Admission type, *n *(%)						< 0.001

Elective	2,656 (15.8)	60 (7.6)^b^	776 (14.2)	888 (16.8)^b^	932 (17.6)^b^	< 0.001

Emergency	13,342 (79.4)	693 (88.2)^b^	4,449 (81.4)	4,137 (78.4)^b^	4,063 (76.9)^b^	< 0.001

Urgent	814 (4.8)	33 (4.2)	238 (4.4)	251 (4.8)	292 (5.5)^b^	0.004

ICU First Service, *n *(%)						< 0.001

CCU	2,944 (17.5)	95 (12.1)^b^	889 (16.3)	1,016 (19.3)^b^	944 (17.9)^b^	< 0.001

CSRU	3,885 (23.1)	78 (9.9)^b^	1,123 (20.6)	1,395 (26.4)^b^	1,289 (24.4)^b^	< 0.001

MICU	5,657 (33.7)	399 (50.8)^b^	1,907 (34.9)	1,566 (29.7)^b^	1,785 (33.8)	< 0.001

SICU	4,326 (25.7)	214 (27.2)	1,544 (28.3)	1,299 (24.6)^b^	1,269 (24.0)^b^	< 0.001

SAPS,^c ^mean (SD)	12.1 (5.3)	12.3 (5.3)	12.2 (5.4)	12.0 (5.3)^b^	12.0 (5.3)^b^	0.87

Smoker, *n *(%)^d^	5,647 (33.9)	256 (32.5)	1,752 (32.1)	1,831 (34.7)^b^	1,808 (34.2)	0.25

Obesity-related conditions						

Diabetes	4,334 (25.8)	117 (14.9)^b^	1,062 (19.4)	1,300 (24.6)^b^	1,855 (35.1)^b^	< 0.001

Coronary artery disease	6,612 (39.3)	182 (23.2)^b^	1,962 (35.9)	2,330 (44.2)^b^	2,138 (40.4)^b^	< 0.001

Stroke	2,014 (12.0)	92 (11.7)	715 (13.1)	643 (12.2)	564 (10.7)^b^	< 0.001

Hypertension	8,647 (51.4)	311 (39.6)^b^	2,516 (46.1)	2,827 (53.6)^b^	2,993 (56.6)^b^	< 0.001

Other CVD	10,066 (59.9)	433 (55.1)^b^	3,257 (59.6)	3,208 (60.8)	3,168 (59.9)	0.16

Obesity-related cancer^e^	384 (2.3)	20 (2.5)	130 (2.4)	115 (2.2)	119 (2.3)	0.54

Kidney disease	4,118 (24.5)	200 (25.5)	1,271 (23.3)	1,234 (23.4)	1,413 (26.7)^b^	< 0.001

Osteoarthritis	280 (1.7)	7 (0.9)	53 (1.0)	92 (1.7)^b^	128 (2.4)^b^	< 0.001

Other relevant diagnoses, *n *(%)						

Pulmonary embolism	328 (2.0)	16 (2.0)	79 (1.5)	101 (1.9)	132 (2.5)^b^	< 0.001

Sepsis	1,194 (7.1)	73 (9.3)^b^	369 (6.8)	353 (6.7)	399 (7.6)	0.60

Wound/skin infection	733 (4.4)	26 (3.3)	186 (3.4)	197 (3.7)	324 (6.1)^b^	< 0.001

ICU interventions, *n *(%)						

Dialysis	1,322 (7.9)	56 (7.1)	417 (7.6)	373 (7.1)	476 (9.0)^b^	0.007

Ventilation	11,201 (66.6)	469 (59.7)^b^	3,574 (65.4)	3,551 (67.3)^b^	3,607 (68.2)^b^	< 0.001

Insulin	6,533 (38.9)	194 (24.7)^b^	1,912 (35.0)	2,198 (41.7)^b^	2,229 (42.2)^b^	< 0.001

Transfusion	6,157 (36.6)	276 (35.1)	2,035 (37.3)	1,987 (37.7)	1,859 (35.2)^b^	0.09

TPN	1,059 (6.3)	59 (7.5)	346 (6.3)	320 (6.1)	334 (6.3)	0.56

Length of stay						

ICU LOS, median (Q1-Q3)	2.3 (1.2-4.8)	2.3 (1.1-4.4)	2.3 (1.2-4.8)	2.3 (1.2-4.7)	2.3 (1.2-4.9)	0.11

Hospital LOS, median (Q1-Q3)	8 (4-13)	8 (4-14)	8 (5-13)	7 (4-13)	8 (5-14)	0.97

Mortality^f^						

Hospital mortality, *n *(%)	2,047 (12.2)	148 (18.8)^b^	799 (14.6)	551 (10.4)^b^	549 (10.4)^b^	< 0.001

Mortality 30 days, *n *(%)	2,339 (13.9)	185 (23.5)^b^	910 (16.7)	644 (12.2)^b^	600 (11.4)^b^	< 0.001

Mortality one year, *n *(%)	4,392 (26.1)	363 (46.2)^b^	1,720 (31.5)	1,203 (22.8)^b^	1,106 (20.9)^b^	< 0.001

^a^*P *value for association or trend across BMI categories. ^b^*P *< 0.05 compared with normal weight. ^c^SAPS was calculated without the age component because age was an independent variable in the analysis. ^d^If smoker status was unknown, they were treated as "no." However, trends were the same when looking at those with data (known status). ^e^Obesity-related cancers: breast, colon, uterine, esophageal, pancreatic, ovarian, and kidney. ^f^The mortality numbers are cumulative (that is, the 1-year mortality group includes the 30-day mortality group. Likewise, the 30-day mortality group includes most of the patients who died in the hospital; however, the few patients that died in the hospital after 30 days were not included in the 30-day mortality.

Clinical covariates included hospitalization characteristics, chronic conditions, and crude mortality by BMI category. Without adjusting for any covariates, patient obesity was associated with a higher incidence of chronic health conditions, including diabetes, hypertension, CAD, and osteoarthritis (*P *< 0.001), but not with a higher rate of obesity-related cancers (*P *= 0.54) compared with normal-weight patients. The mean SAPS were very similar across BMI categories, ranging from 12.0 to 12.3. As might be expected, overweight and obese patients were more often mechanically ventilated, treated with insulin, and more likely to develop wound and skin infections as well as pulmonary embolism (*P *< 0.001 for each). No significant trend was noted for either ICU or hospital length of stay across BMI categories. The crude in-hospital, 30-day, and 1-year mortality rates for overweight and obese patients were significantly lower compared with those of normal-weight patients (*P *< 0.001 for each). In general, although some differences existed between the BMI groups with respect to various covariates, none of the differences seem to be large enough or to be of sufficient clinical importance to account for the effect of BMI on mortality.

**Figure 2:**
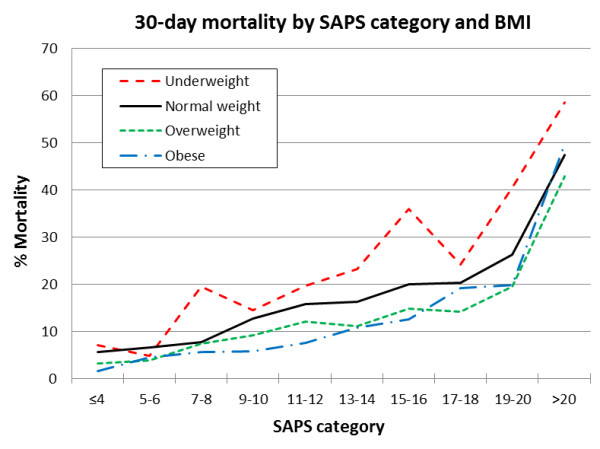
**The 30-day mortality by SAPS category and BMI**. Graph illustrating the differences in 30-day mortality based on the admission SAPS score and BMI category.

As part of the univariate analysis, we calculated mortality rates by BMI within each SAPS category and found that for the same level of admission acuity (that is, the same SAPS category), overweight and obese patients generally had a lower mortality rate compared with those with normal weight (see Figure 2), and most of these results were statistically significant (see Table 3). Only in the highest SAPS category (the sickest patients) was the mortality of obese patients greater than that of the normal-weight patients, but this difference was not statistically significant. We adjusted for SAPS in our final model.

**Table 3:** Univariate analysis of Initial SAPS distribution and 30-day mortality by body mass index category.

	Total (16,812)		Underweight (786)		Normal (5,463)		Overweight (5,276)		Obese (5,287)	
**SAPS category (maximum possible, 56)**	***n *(%)**	**Mortality %**	***n *(%)**	**Mortality %**	***n *(%)**	**Mortality %**	***n *(%)**	**Mortality %**	***n *(%)**	**Mortality %**

≤ 4	1,152 (6.9%)	3.6%	42 (5.3%)	7.1%	356 (6.5%)	5.6%	387 (7.3%)	3.1%	367 (6.9%)	1.6%^a^

5-6	1,425 (8.5%)	4.9%	63 (8.0%)	4.8%	440 (8.1%)	6.6%	449 (8.5%)	3.8%	473 (8.9%)	4.4%

7-8	1,969 (11.7%)	7.4%	93 (11.8%)	19.4%^a^	648 (11.9%)	7.6%	593 (11.2%)	7.3%	635 (12.0%)	5.5%

9-10	2,193 (13.0%)	9.7%	117 (14.9%)	14.5%	731 (13.4%)	12.7%	694 (13.2%)	9.2%^a^	651 (12.3%)	5.8%^a^

11-12	2,362 (14.0%)	12.1%	122 (15.5%)	19.7%	754 (13.8%)	15.7%	713 (13.5%)	12.1%^a^	773 (14.6%)	7.5%^a^

13-14	2,321 (13.8%)	13.1%	95 (12.1%)	23.2%	732 (13.4%)	16.3%	740 (14.0%)	11.0%^a^	754 (14.3%)	10.7%^a^

15-16	2,016 (12.0%)	16.6%	92 (11.7%)	35.9%^a^	629 (11.5%)	19.9%	672 (12.7%)	14.7%^a^	623 (11.8%)	12.5%^a^

17-18	1,511 (9.0%)	18.1%	62 (7.9%)	24.2%	493 (9.0%)	20.3%	473 (9.0%)	14.2%^a^	483 (9.1%)	19.1%

19-20	862 (5.1%)	23.1%	47 (6.0%)	40.4%^a^	309 (5.7%)	26.2%	268 (5.1%)	19.4%	238 (4.5%)	19.8%

> 20 (21-35)^b^	1,001 (6.0%)	47.4%	53 (6.7%)	58.5%	371 (6.8%)	47.4%	287 (5.4%)	42.9%	290 (5.5%)	49.7%

^a^*P *< 0.05 compared with normal weight. ^b^We followed the original SAPS study, which grouped all of the patients with SAPS > 20 in one category, and the maximum SAPS we observed was 35.

To account for conditions that might affect acuity that the SAPS score does not account for, we analyzed the distribution of 30-day mortality by BMI category and significant underlying diseases at admission based on comorbid conditions that are part of the SAPS II acuity score. Results are shown in Table 4. Twenty-five percent of obese patients with metastatic cancer died within 30 days compared with 36% of normal-weight patients with metastatic cancer, which is consistent with our hypothesis that obesity is protective.

**Table 4:** Univariate analysis of the distribution of significant underlying diseases and 30-day mortality by body mass index category.

	Total (16,812)		Underweight (786)		Normal (5,463)		Overweight (5,276)		Obese (5,287)	
	
Diagnosis (ICD-9 code(s))	*n *(%)	Mortality %	*n *(%)	Mortality %	*n *(%)	Mortality %	*n *(%)	Mortality %	*n *(%)	Mortality %
HIV (042)	195 (1.1%)	14.9%	36 (4.6%)	8.3%	102 (1.9%)	13.7%	42 (0.8%)	23.8%	15 (0.3%)	13.3%
Leukemia/lymphoma/multiple myeloma (200-208)	449 (2.7%)	28.5%	30 (3.8%)	40.0%	160 (2.9%)	31.3%	134 (2.5%)	21.6%	125 (2.4%)	29.6%
Metastatic cancer (196-198)	911 (5.4%)	30.9%	61 (7.8%)	31.2%	350 (6.6%)	36.3%	251 (4.8%)	28.7%	249 (4.7%)	25.3%^a^

^a^*P *< 0.05 compared with normal weight.

The univariate logistic regression models estimated crude mortality risk 30 days and 1 year after ICU admission by BMI category without adjusting for any covariates. Both the overweight and obese patients had markedly lower mortality risk at both 30 days (OR, 0.70; 95% CI, 0.62 to 0.78) and 0.64 (95% CI, 0.57 to 0.72, respectively) and 1 year (OR, 0.64 (95% CI, 0.59 to 0.70) and 0.58 [95% CI, 0.53-0.63]) compared with normal-weight patients. In contrast, underweight patients had significantly higher risk in both time periods (30 days: OR, 1.54 (95% CI, 1.29 to 1.84); 1 year: OR, 1.87 (95% CI, 1.61 to 2.17)).

**Figure 3:**
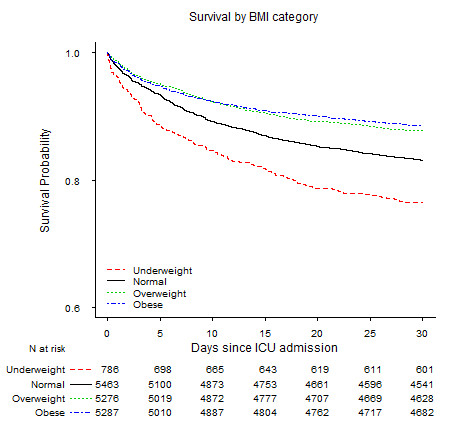
**Kaplan-Meier curve for 30-day survival by BMI category**. Kaplan-Meier curve for survival by BMI category over the first 30-day period. The numbers represent the number of patients still surviving in each BMI category at the start of each time period. Note: the survival probability is truncated at 0.6 for display purposes.

Our multivariable regression models revealed relations between BMI and mortality that were very similar to those of the univariate regression analyses (see Table 5). Adjusting for age, sex, race, and obesity-related conditions such as diabetes, CAD, and kidney disease to the multivariable model did not appreciably change the mortality risk for any BMI category compared with the univariate regression analysis. Even after adjusting for all of the clinical covariates in Table 5, our results were remarkably similar to the univariate risk estimates: the overweight and obese groups had mortality risks that were 19% and 26% lower at 30 days (OR, 0.81 (95% CI, 0.70 to 0.93) and 0.74 (95% CI, 0.64 to 0.86)) and an additional 13% and 17% lower at 1 year, respectively (OR, 0.68 (95% CI, 0.59 to 0.79) and 0.57 (95% CI, 0.49 to 0.67)) compared with normal-weight patients. Underweight patients had 40% and 50% higher mortality risk at 30 days and 1 year (OR, 1.41 (95% CI, 1.13 to 1.76) and 1.51 (95% CI, 1.18 to 1.94)). The Kaplan-Meier survival curves, shown in Figure 3, clearly show the same survival advantage for overweight and obese patients over time (log-rank test, *P *< 0.001).

**Table 5:** Multivariable logistic regression results for 30-day and one-year mortality risk.

	30 days Odds ratio (95% CI)	1 year Odds oatio (95% CI)
BMI category (ref, normal weight (18.5 to < 25 kg/m^2^))		

Underweight (< 18.5 kg/m^2^)	1.41 (1.13, 1.76)^b^	1.51 (1.18, 1.94)^b^

Overweight (25 to < 30 kg/m^2^)	0.81 (0.70, 0.93)^b^	0.68 (0.59, 0.79)^b^

Obese (30 to < 40 kg/m^2^)	0.74 (0.64, 0.86)^b^	0.57 (0.49, 0.67)^b^

Age category (ref < 45 years)		

45 to < 65 years	2.44 (1.93, 3.07)^b^	2.56 (2.01, 3.28)^b^

65 to < 80 years	3.91 (3.08, 4.96)^b^	4.43 (3.43, 5.72)^b^

80+ years	8.16 (6.39, 10.43)^b^	7.51 (5.72, 9.85)^b^

Female	0.91 (0.81, 1.02)	0.87 (0.77, 0.99)^b^

Race (ref, White)		

Black	0.60 (0.48, 0.75)^b^	0.69 (0.55, 0.86)^b^

Hispanic or Latino	0.53 (0.35, 0.81)^b^	0.48 (0.31, 0.75)^b^

Asian	0.46 (0.30, 0.69)^b^	0.89 (0.61, 1.30)

Other	1.15 (0.82, 1.63)	1.21 (0.83, 1.76)

Insurance (ref, CMS)		

Private	0.85 (0.74, 0.98)^b^	0.83 (0.72, 0.95)^b^

Other	0.91 (0.71, 1.17)	0.63 (0.46, 0.86)^b^

Smoker	a	1.26 (1.11, 1.43)^b^

Obesity-related conditions		

Diabetes	0.82 (0.71, 0.94)^b^	a

Coronary artery disease	0.68 (0.59, 0.78)^b^	0.65 (0.56, 0.75)^b^

Stroke	2.15 (1.84, 2.51)^b^	1.44 (1.21, 1.73)^b^

Hypertension	0.64 (0.56, 0.72)^b^	0.58 (0.51, 0.66)^b^

Other CVD	a	1.47 (1.27, 1.69)^b^

Obesity-related cancer^c^	1.38 (1.00, 1.92)	1.89 (1.36, 2.62)^b^

Kidney disease	1.50 (1.32, 1.71)^b^	1.94 (1.70, 2.21)^b^

Osteoarthritis	0.28 (0.15, 0.54)^b^	0.39 (0.23, 0.66)^b^

Other relevant diagnoses		

Sepsis	1.56 (1.32, 1.85)^b^	1.51 (1.24, 1.82)^b^

Pulmonary embolism	2.06 (1.47, 2.88)^b^	a

ICU Unit (ref, MICU)		

CCU	0.76 (0.63, 0.92)^b^	0.62 (0.51, 0.74)^b^

CSRU	0.15 (0.12, 0.19)^b^	0.17 (0.13, 0.21)^b^

SICU	0.66 (0.57, 0.77)^b^	0.73 (0.62, 0.85)^b^

SAPS^d^	1.18 (1.16, 1.19)^b^	1.09 (1.07, 1.10)^b^

ICU interventions		

Ventilation	1.36 (1.17, 1.59)^b^	a

Transfusion	0.74 (0.65, 0.84)^b^	1.18 (1.04, 1.34)^b^

TPN	0.85 (0.68, 1.05)	a

^a^Dropped out of the final multivariable model. ^b^*P *< 0.05. ^c^Obesity-related cancers: breast, colon, uterine, esophageal, pancreatic, ovarian, and kidney. ^d^SAPS was calculated without the age component because age was an independent variable in the analysis.

As expected, older age and higher admission SAPS were associated with increased mortality risk in both time periods (*P *< 0.05). Racial minorities had a lower risk of mortality; however, each minority group represented < 10% of the study population, whereas the reference group comprised 67%. Patients in the SICU and CCU had a 34% and 24% lower mortality risk, respectively, at 30 days (OR, 0.66 (95% CI, 0.57 to 0.77) and 0.76 (95% CI, 0.63 to 0.92)) and 27% and 38% lower risk at 1 year (OR, 0.73 (95% CI, 0.62 to 0.85) and 0.62 (95% CI, 0.51 to 0.74)) compared with the MICU patients, and CSRU patients had a markedly lower risk in both time periods (OR, 0.15 (95% CI, 0.12 to 0.19) and 0.17 (95% CI, 0.13 to 0.21)). The survival advantage for patients in the CSRU is not surprising, given that patients admitted to the CSRU are preselected for elective cardiac surgery, whereas those in the MICU are generally admitted as an emergency with more critical diagnoses.

As expected, high-mortality conditions, including obesity-related cancers, kidney disease, stroke, and sepsis, were associated with increased mortality risk in both time periods. Pulmonary embolus was associated with higher risk at 30 days, and other CVD was associated with higher risk at 1 year (*P *< 0.05 for each). Likewise, lower-acuity conditions, including CAD, hypertension, and osteoarthritis, were associated with lower risk at both 30 days and 1 year (*P *< 0.05). Interestingly, patients with diabetes had lower risk at 30 days (OR, 0.82 (95% CI, 0.71 to 0.94)) but dropped out of the final regression model for 1-year mortality risk.

### Sensitivity and Cox proportional hazards analyses

Our results did not change appreciably in any of our sensitivity analyses. When we removed the patients with imputed heights, the results of the final multivariable regression model remained consistent with the primary analysis in which those with imputed heights were included. We also compared the 2,179 patients without any weight measurements with the 16,812 included in our final analysis. Patients without weight data were more likely to be younger, admitted to the MICU or SICU, have shorter ICU and hospital stays, and had a lower mortality rate at both 30 days and 1 year after ICU admission. Patients without weight data had better outcomes, and for this reason, we imputed "normal" BMIs for these patients because this assumption was most likely to reverse the mortality advantages of overweight and obese patients. We then reran the final models, and the results did not change. At 30 days, the overweight and obese patients had a mortality risk of 0.78 (95% CI, 0.59 to 0.79) and 0.72 (95% CI, 0.62 to 0.82), respectively, compared with 0.81 (95% CI, 0.70 to 0.93) and 0.74 (95% CI, 0.64 to 0.86) when the patients with missing weights were not included; at 1 year, the odds ratios for mortality risk were exactly the same for both the overweight and obese groups, whether or not the patients with the missing weights were included in the normal-BMI group.

The results of the analyses without the underweight group did not change the primary results for the normal, overweight, or obese groups. When examined as a separate group, the severely obese patients (BMI ≥ 40 kg/m^2^; *n *= 1,119) were similar to the obese group as a whole, in that they were more likely to be younger, have private insurance, and have a higher incidence of diabetes, hypertension, kidney disease, and osteoarthritis compared with normal-weight patients. In contrast to the obese group as a whole, the severely obese patients were more likely to be female and have a slightly longer median ICU length of stay, and they did not have lower 30-day mortality risk than did normal-weight patients. However, they still had significantly lower 1-year risk compared with normal-weight patients (OR, 0.70 (95% CI, 0.54 to 0.90)). After taking out the severely obese, the obese group (BMI 30 to < 40 kg/m^2^) had an additional 3% to 5% reduction in risk at both time periods. (See Additional File 1 for detailed results for the severely obese group.)

To address potential collinearity in the four CVD predictors in our final model, we reran the final model by using a more comprehensive, composite CVD variable that included the four separate cardiac variables in addition to other CVD conditions. This model produced similar results for mortality risk by BMI category.

When we examined patients in each ICU unit separately, the association between BMI category and mortality at both 30 days and 1 year was directionally the same within each ICU unit compared with the estimate in the final model.

We analyzed each obesity-related condition (diabetes, CAD, stroke, hypertension, other CVD, obesity-related cancers, kidney disease, and osteoarthritis), admission SAPS, and ICU intervention (ventilation, transfusion, and TPN) to see if any of these potential confounders mediated the relation between BMI category and mortality risk. The largest effect that any of the obesity-related conditions had on any of the four BMI categories was CAD on the underweight BMI group, but this effect was neither significant nor clinically important (the OR for mortality risk decreased from 1.64 (95% CI, 1.34 to 2.0) to 1.53 (95% CI, 1.25 to 1.88) at 30 days and 1.94 (95% CI, 1.63 to 2.31) to 1.83 (95% CI, 1.54 to 2.18) at 1 year). Neither the admission SAPS nor the ICU interventions had any significant effect (0 to 4% attenuation across BMI categories in both time periods). When we analyzed β-blockers, statins, and insulin as potential confounders, we found that they were also not clinically significant, and adding them to the model resulted in less than 2% attenuation in both time periods (none of the ORs changed by > 0.01).

Last, the estimates of risk from the Cox proportional hazard models were consistent with the final logistic regression models with both methods using the same predictors, indicating that the results were robust to changes in statistical methods.

## Discussion

Critically ill patients who were obese or overweight had markedly lower 30-day and 1-year mortality risk despite having higher incidences of many comorbidities and similar admission acuity compared with their normal-weight counterparts. At 1 year after critical illness, even severely obese patients had a significant survival benefit. The protective effect of obesity (termed the "obesity paradox") has also been reported in obese patients with specific diagnoses, including acute heart failure [31], chronic kidney disease [32], and HIV/AIDS [33].

Is a physiological explanation known for this apparent survival advantage of obesity in critical care? Animal studies have shown that increases in adipocyte size in obesity lead to M1 macrophage accumulation in adipose tissue, which produce proinflammatory cytokines and are linked to insulin resistance [34,35]. In contrast, critically ill patients have a higher percentage of newly formed, smaller adipocytes compared with healthy controls [36], and these adipocytes contain large accumulations of alternatively activated M2 macrophages that produce higher levels of a number of antiinflammatory agents that increase insulin sensitivity and promote wound healing [37]. Human macrophages have been shown to switch between the M1 and M2 phenotypes during critical illness [38], so perhaps one mechanism by which overweight and obesity are protective during critical illness may be a switch from M1 proinflammatory activation to alternative M2 antiinflammatory activation in the large population of macrophages that are already present in their adipose tissue compared with normal-weight individuals.

Another theory is the protection provided by increased nutritional reserves during times of illness [16,19]. Although normal-weight individuals have adequate nutritional intake to cover daily metabolic requirements, they may not have the stores necessary to sustain organ function during a time of significantly increased metabolic demand, such as critical illness. A recent study by Alberda and colleagues [39] focused on the importance of nutritional supplementation for critically ill patients, and they found that the positive effect of increased nutrition occurred primarily in under- and normal-weight patients and a small subset of middle-range obese patients [39].

Others hypothesized that obese patients might be admitted to the ICU for less-acute illness because of increased nursing needs and that they fare better because they are initially less sick [20]; however, in our study, the initial mean SAPS was strikingly similar across BMI categories (see Table 2). Furthermore, overweight and obese patients had lower mortality compared with the normal-weight patients in almost every SAPS category. The exception to this was the obese patients in the highest (SAPS > 20) category, whose mortality rate was similar to that of the normal-weight patients in that category and whose prior risk could have overwhelmed the putative advantage of obesity.

Each of the four ICU types had different patient populations in terms of acuity, diagnoses, and interventions, but our results still held when we analyzed the patients in each ICU unit separately. We found that the direction of association between BMI category and mortality remained the same within each ICU category, although not all were significant, given the smaller sample sizes when we divided patients by ICU. In other words, despite the inherent differences between the patients in the MICU, SICU, CCU, and CSRU, obese and overweight patients had a lower mortality risk, and underweight patients had a higher risk compared with normal-weight patients within each type of ICU as well as across ICUs.

To assess baseline health status before admission, we determined whether patients had one of the following diagnoses: HIV, leukemia/lymphoma/multiple myeloma, and metastatic cancer. The blood disorders and metastatic cancer diagnoses were fairly evenly distributed across the normal, overweight, and obese patients, which was consistent with the distribution of our overall study population. Far fewer overweight and obese patients had HIV compared with normal weight, but HIV patients composed only 1% of our study population. A significantly smaller percentage of obese patients with metastatic cancer died within 30 days compared with normal-weight patients with metastatic cancer, which supports the protective effect we hypothesize. The results for HIV and leukemia/lymphoma do not show the same relation and were not statistically significant; however, the number of patients having these diseases was also small, with little power to show significant differences.

As others have noted [20], we found that obese patients admitted to the ICU tended to be younger compared with normal and underweight patients. We also found that obese and overweight patients made up most (68%) of those covered by private insurance rather than Medicare or Medicaid, which are state or federal programs in the United States that provide insurance for the elderly or patients with low income or significant chronic health conditions. Both the age and insurance differences could reflect intrinsic differences in the general health or access to healthcare and might explain our results; however, the survival advantage of obesity and overweight compared with normal weight persisted when we controlled for age and type of insurance in our multivariable models.

As discussed previously, many of the prior studies did not compare outcomes based on the standard WHO categories. Critically ill underweight patients are known to have higher mortality [3,40,41] compared with normal-weight patients. The studies that combined the underweight patients with the normal-weight patients [9,14] may have masked the true relation between mortality and BMI category. Results from those studies that compared obese patients with a single group that included normal, underweight, and overweight patients [7,8,11,12] are even more difficult to interpret. Furthermore, that bias could have carried over to the results of both meta-analyses, which were based on several of these same studies. Our investigation clearly separated the underweight, normal-weight, overweight, and obese patients into the standard WHO BMI categories, and the BMI distribution of our study population generally reflects that of the general adult U.S. population [1]. In addition, the mortality advantage of overweight and obesity persisted even when we excluded the underweight patients from our analysis entirely, and severely obese patients by themselves also had a significant advantage at 1 year compared with normal-weight patients.

Height measurements were missing for 25% of the adults with weight values in the MIMIC-II database. In the ICU setting, measuring height, which requires patients to stand, can be impossible, given the patient's critical state and likely connection to tubes and ventilators. In addition, patients admitted for observation for short periods may not have heights measured because their height value may not be relevant to their ICU stay. We chose to include the patients lacking height measurements by assigning them imputed height values. Other studies have lacked greater proportions of such data but simply excluded them [15]. We checked for potential bias by removing the patients with imputed heights and running the model again, and we found no important differences in the effect of obesity on survival.

For the 11% of adult patients in MIMIC-II that were missing values for body weight, we took a different approach and excluded them from the primary model. We did this because population weights vary more than population heights, which makes imputation less reliable, and the total number missing was much smaller. Patients without weights were significantly younger, had shorter lengths of stay, and a lower ICU mortality rate. To be sure that the exclusion of patients who were missing weights did not distort our conclusions, in a subsequent sensitivity analysis, we included these patients in the normal-BMI category, because putting the patients with good survival into the normal-weight group was most likely to change our conclusions. But, as detailed in the Results, the outcome was almost exactly the same whether or not they were included and continued to support the protective effect of obesity.

For patients in the highest SAPS category (> 20), the mortality rate was much higher compared with that of patients in the second-highest SAPS category (19-20), and no significant differences in mortality were found between BMI groups. This finding suggests that the advantage of overweight and obesity might be attenuated for the most critically ill patients. This observation might be relevant in countries where ICU beds are reserved for more critical patients than in the United States. For example, a recent study compared ICU care in the United States and the United Kingdom and found that the admission APACHE score was significantly higher in the U.K. versus the U.S. [42]. The higher acuity score in the U.K. was a reflection of ICU-bed availability: one seventh of the number of ICU beds per capita are available in the U.K. compared with the U.S., so only the sickest patients are admitted to the ICU.

MIMIC-II offered many advantages for our analysis, including large sample size, a variety of admission types and diagnoses, and long-term death data. However, our study shares the disadvantages of most retrospective studies, including missing data issues [43], lack of randomization, and a potential for model misspecification. The most important missing data element in our study was patient height, but as described earlier, the sensitivity analysis showed that including the patients with imputed heights did not change the results. Regarding study design, as one cannot randomize patients to BMI categories, only observational studies of BMI and mortality are possible, so we have to adjust for confounding through other means, such as regression. With regard to mis-specification, although it is possible to exclude inadvertently an important confounder from a statistical model, we chose a comprehensive set of variables based on clinical experience as well as the current literature, and MIMIC-II contained all of the variables we wished to include in the model.

A common issue across clinical databases is underreporting of patient diagnoses by using ICD-9 billing codes. Most of our comorbidity data were drawn from the billing table, and underreporting of nonacute comorbid conditions may have occurred because they are not the focus of a hospitalization and/or they do not contribute to reimbursed diagnosis-related group codes (DRG). This may explain the "protective" odds ratios we found for diabetes, CAD, hypertension, and osteoarthritis.

Finally, we would have liked to comment on changes in the obesity rate and trends in ICU care over time, but we were unable to do so because of the random date shifting that MIMIC-II used as part of its deidentification process.

## Conclusions

Our study found that overweight and obese patients have a much lower mortality risk after ICU admission. Although the exact mechanism of the association is not known, we performed extensive analyses to assure that our findings were not due to confounding factors or other biases. Two potential remaining explanations are the benefits of adipose tissue in terms of (a) alternative macrophage activation and beneficial antiinflammatory agents, and (b) greater nutritional reserves. We found that even after adjusting for multiple potential risk factors, including age, acuity, obesity-related diseases, and hospital interventions, overweight and obesity still had a substantial beneficial effect on survival for at least 1 year after ICU admission.

## Key messages

• Overweight and obese patients have significantly lower mortality both 30 days and 1 year after ICU admission.

• The "protective effect" of overweight and obesity holds true after adjusting for multiple factors, including demographics, admission SAPS score, ICU unit, obesity-related conditions, and various ICU interventions.

• Alternatively activated macrophages may play a role in lowering mortality risk after an ICU admission for overweight and obese patients.

## Abbreviations

BMI: Body mass index; CAD: coronary artery disease; CCU: cardiac care unit; CSRU: cardiac surgery recovery unit; CVD: cardiovascular disease; ICD-9: International Classification of Diseases, 9^th ^revision; ICU: intensive care unit; MICU: medical intensive care unit; MIMIC-II: Multiparameter Intelligent Monitoring in Intensive Care; MIT: Massachusetts Institute of Technology; OR: odds ratio; PE: pulmonary embolus; SAPS: Simplified Acute Physiology Score; SICU: surgical intensive care unit; TPN: total parenteral nutrition; VIF: variance inflation factor; WHO: World Health Organization.

## Competing interests

None of the authors has any financial or nonfinancial competing interests to declare.

## Authors' contributions

SA participated in study conception and design, data acquisition, data interpretation, drafting of the manuscript, and critical revisions of the manuscript. FC participated in study conception and design, data acquisition, statistical analysis and data interpretation, and critical revisions of the manuscript. DD participated in study conception and design, data acquisition, data interpretation, and critical revisions of the manuscript. KL participated in study conception and design, data acquisition, statistical analysis and data interpretation, drafting of the manuscript, and critical revisions of the manuscript. CJM participated in study conception and design, data interpretation, and critical revisions of the manuscript, and also supervised the study. All of the authors read and approved this manuscript for publication.

## Supplementary Material

Additional file 1 **Univariate analysis tables with the severely obese patients as a separate group**. Additional file 1 includes four tables with results of the univariate analyses after splitting the severely obese patients (body mass index, ≥ 40 kg/m^2^) from the rest of the obese patients (body mass index, 30 to < 40 kg/m^2^). Table S1 is the univariate analysis of demographic characteristics. Table S2 is the univariate analysis of hospitalization characteristics, chronic conditions, and mortality. Table S3 is the univariate analysis of the initial SAPS distribution and 30-day mortality. Table S4 is the univariate analysis of the distribution and 30-day mortality of significant underlying illnesses. Click here for file
