# Supplementary material for: Lower short- and long-term mortality associated with overweight and obesity in a large cohort study of adult intensive care unit patients
Source: Crit Care. 2012 Dec 18;16(6):R235. doi: 10.1186/cc11903 (PMC3672624; doi:10.1186/cc11903)
Supplement: Additional file 1 — Univariate analysis tables with the severely obese patients as a separate group. Additional file 1 includes four tables with results of the univariate analyses after splitting the severely obese patients (body mass index, ≥ 40 kg/m2) from the rest of the obese patients (body mass index, 30 to < 40 kg/m2). Table S1 is the univariate analysis of demographic characteristics. Table S2 is the univariate analysis of hospitalization characteristics, chronic conditions, and mortality. Table S3 is the univariate analysis of the initial SAPS distribution and 30-day mortality. Table S4 is the univariate analysis of the distribution and 30-day mortality of significant underlying illnesses. [file cc11903-S1.DOC]

| **Table S1.** Univariate analysis of demographic characteristics by body mass index (BMI) category with the severely obese patients examined as a separate group. | | | | | | | |
| --- | --- | --- | --- | --- | --- | --- | --- |
|  | **Total** | **Underweight**  **(BMI <18.5 kg/m^2^)** | **Normal**  **(BMI 18.5-<25 kg/m^2^)** | **Overweight (BMI 25-<30 kg/m^2^)** | **Obese (BMI 30-<40 kg/m^2^)** | **Severely obese (BMI ≥40 kg/m^2^)** | **p-value^a^** |
| **N (%)** | 16812 | 786 (4.7) | 5463 (32.5) | 5276 (31.4) | 4168 (24.8) | 1119 (6.7) |  |
| **Age (years), N (%)** |  |  |  |  |  |  | <0.001 |
| <45 | 2522 (15.0) | 118 (15.0) | 905 (16.6) | 757 (14.4) ^b^ | 551 (13.2) ^b^ | 191 (17.1) | 0.003 |
| 45-65 | 5593 (33.3) | 219 (27.9) | 1467 (26.9) | 1677 (31.8) ^b^ | 1681 (40.3) ^b^ | 549 (49.1) ^b^ | <0.001 |
| 65-80 | 5393 (32.1) | 215 (27.4) | 1673 (30.6) | 1814 (34.4) ^b^ | 1397 (33.5) ^b^ | 294 (26.3) ^b^ | 0.05 |
| 80+ | 3304 (19.7) | 234 (29.8)^b^ | 1418 (26.0) | 1028 (19.5) ^b^ | 539 (12.9) ^b^ | 85 (7.6) ^b^ | <0.001 |
| **Age (years), median (Q1-Q3)** | 65.9 (52.3, 77.8) | 70.6 (53.0, 81.8) | 69.4 (51.7, 80.4) | 67.2 (53.6, 77.8) | 63.4 (52.6, 74.2) ^b^ | 59.1 (49.3, 68.9) ^b^ | <0.001 |
| **Female, N (%)** | 7170 (42.7) | 443 (56.4) ^b^ | 2469 (45.2) | 1901 (36.0) ^b^ | 1702 (40.8) ^b^ | 655 (58.5) ^b^ | 0.02 |
| **Race, N (%)** |  |  |  |  |  |  | <0.001 |
| White | 11390 (82.9) | 534 (77.3) ^b^ | 3667 (81.5) | 3606 (84.3) ^b^ | 2826 (84.9) ^b^ | 757 (79.9) | <0.001 |
| Black | 1179 (8.6) | 71 (10.3) ^b^ | 355 (7.9) | 333 (7.8) | 280 (8.4) | 140 (14.8) ^b^ | 0.002 |
| Hispanic or Latino | 430 (3.1) | 16 (2.3) | 148 (3.3) | 144 (3.4) | 94 (2.8) | 28 (3.0) | 0.60 |
| Asian | 322 (2.3) | 47 (6.8) ^b^ | 173 (3.8) | 72 (1.7)* | 24 (0.7) ^b^ | 6 (0.6) ^b^ | <0.001 |
| Other | 424 (3.1) | 23 (3.3) | 157 (3.5) | 121 (2.8) | 106 (3.2) | 17 (1.8) ^b^ | 0.05 |
| **Marital status, N (%)** |  |  |  |  |  |  | <0.001 |
| Married | 8317 (53.7) | 271 (38.6) ^b^ | 2524 (50.6) | 2819 (57.8) ^b^ | 2198 (56.9) ^b^ | 505 (48.1) | <0.001 |
| Single/Divorced/ Separated | 4690 (30.3) | 278 (39.5) ^b^ | 1545 (31.0) | 1359 (27.9) ^b^ | 1110 (28.7) ^b^ | 398 (37.9) ^b^ | 0.11 |
| Widowed | 2479 (16.0) | 154 (21.9) ^b^ | 923 (18.5) | 699 (14.3) ^b^ | 556 (14.4) ^b^ | 147 (14.0) ^b^ | <0.001 |
| **Insurance, N (%)** |  |  |  |  |  |  | <0.001 |
| Medicare/Medicaid^b^ | 8806 (52.4) | 501 (63.7) ^b^ | 3091 (56.6) | 2658 (50.4) ^b^ | 2000 (48.0) ^b^ | 556 (49.7) ^b^ | <0.001 |
| Private | 6724 (40.0) | 231 (29.4) ^b^ | 1877 (34.4) | 2221 (42.1) ^b^ | 1896 (45.5) ^b^ | 499 (44.6) ^b^ | <0.001 |
| Other | 1282 (7.6) | 54 (6.9) ^b^ | 495 (9.1) | 397 (7.5) ^b^ | 272 (6.5) ^b^ | 64 (5.7) ^b^ | <0.001 |
| ^a^p-value for association or trend across BMI categories; ^b^p<0.05 compared to normal weight; ^b^Medicare/Medicaid are state or federal programs in the U.S. that provide insurance for the elderly or patients with low income or significant chronic health conditions | | | | | | | |

| **Table S2.** Univariate analysis of hospitalization characteristics, chronic conditions, and mortality by body mass index (BMI) category with the severely obese patients examined as a separate group. | | | | | | | |
| --- | --- | --- | --- | --- | --- | --- | --- |
|  | **Total** | **Underweight (BMI <18.5** **kg/m^2^)** | **Normal weight (BMI 18.5-<25** **kg/m^2^)** | **Overweight (BMI 25-<30** **kg/m^2^)** | **Obese (BMI 30-<40 kg/m^2^)** | **Severely obese**  **(BMI ≥40 kg/m^2^)** | **p-value^a^** |
| **Admission type, N (%)** |  |  |  |  |  |  | <0.001 |
| Elective | 2656 (15.8) | 60 (7.6)^b^ | 776 (14.2) | 888 (16.8)^b^ | 748 (18.0) ^b^ | 184 (16.4) | <0.001 |
| Emergency | 13342 (79.4) | 693 (88.2)^b^ | 4449 (81.4) | 4137 (78.4)^b^ | 3182 (76.3) ^b^ | 881 (78.7) ^b^ | <0.001 |
| Urgent | 814 (4.8) | 33 (4.2) | 238 (4.4) | 251 (4.8) | 238 (5.7) ^b^ | 54 (4.8) | 0.006 |
| **ICU First Service, N (%)** |  |  |  |  |  |  | <0.001 |
| CCU | 2944 (17.5) | 95 (12.1)^b^ | 889 (16.3) | 1016 (19.3)^b^ | 785 (18.8) ^b^ | 159 (14.2) | 0.002 |
| CSRU | 3885 (23.1) | 78 (9.9)^b^ | 1123 (20.6) | 1395 (26.4)^b^ | 1090 (26.2) ^b^ | 199 (17.8) ^b^ | <0.001 |
| MICU | 5657 (33.7) | 399 (50.8)^b^ | 1907 (34.9) | 1566 (29.7)^b^ | 1292 (31.0) ^b^ | 493 (44.1) ^b^ | <0.001 |
| SICU | 4326 (25.7) | 214 (27.2) | 1544 (28.3) | 1299 (24.6)^b^ | 1001 (24.0) ^b^ | 268 (24.0) ^b^ | <0.001 |
| **SAPS^c^, mean (SD)** | 12.1 (5.3) | 12.3 (5.3) | 12.2 (5.4) | 12.0 (5.3)^b^ | 11.9 (5.2) ^b^ | 12.2 (5.3) | 0.22 |
| **Smoker, N (%)^d^** | 5647 (33.9) | 256 (32.5) | 1752 (32.1) | 1831 (34.7)^b^ | 1421 (34.1) | 387 (34.6) | 0.26 |
| **Obesity-related conditions** |  |  |  |  |  |  |  |
| Diabetes | 4334 (25.8) | 117 (14.9)^b^ | 1062 (19.4) | 1300 (24.6)^b^ | 1413 (33.9) ^b^ | 442 (39.5) ^b^ | <0.001 |
| Coronary Artery Disease | 6612 (39.3) | 182 (23.2)^b^ | 1962 (35.9) | 2330 (44.2)^b^ | 1807 (43.4) ^b^ | 331 (29.6) ^b^ | <0.001 |
| Stroke | 2014 (12.0) | 92 (11.7) | 715 (13.1) | 643 (12.2) | 467 (11.2) ^b^ | 97 (8.7) ^b^ | <0.001 |
| Hypertension | 8647 (51.4) | 311 (39.6)^b^ | 2516 (46.1) | 2827 (53.6)^b^ | 2367 (56.8) ^b^ | 626 (55.9 ^b^) | <0.001 |
| Other CVD | 10066 (59.9) | 433 (55.1)^b^ | 3257 (59.6) | 3208 (60.8) | 2488 (59.7) | 680 (60.8) | 0.14 |
| Obesity-related Cancer^e^ | 384 (2.3) | 20 (2.5) | 130 (2.4) | 115 (2.2) | 91 (2.2) | 28 (2.5) | 0.60 |
| Kidney Disease | 4118 (24.5) | 200 (25.5) | 1271 (23.3) | 1234 (23.4) | 1058 (25.4) ^b^ | 355 (31.7)^b^ | <0.001 |
| Osteoarthritis | 280 (1.7) | 7 (0.9) | 53 (1.0) | 92 (1.7)^b^ | 94 (2.3) ^b^ | 34 (3.0) ^b^ | <0.001 |
| **Other relevant diagnoses, N (%)** |  |  |  |  |  |  |  |
| Pulmonary Embolism | 328 (2.0) | 16 (2.0) | 79 (1.5) | 101 (1.9) | 96 (2.3) ^b^ | 36 (3.2) ^b^ | <0.001 |
| Sepsis | 1194 (7.1) | 73 (9.3)^b^ | 369 (6.8) | 353 (6.7) | 281 (6.7) | 118 (10.6) ^b^ | 0.28 |
| Wound/skin infection | 733 (4.4) | 26 (3.3) | 186 (3.4) | 197 (3.7) | 231 (5.5) ^b^ | 93 (8.3) ^b^ | <0.001 |
| **ICU interventions, N (%)** |  |  |  |  |  |  |  |
| Dialysis | 1322 (7.9) | 56 (7.1) | 417 (7.6) | 373 (7.1) | 341 (8.2) | 135 (12.1) ^b^ | 0.001 |
| Ventilation | 11201 (66.6) | 469 (59.7)^b^ | 3574 (65.4) | 3551 (67.3)^b^ | 2814 (67.5) ^b^ | 793 (70.9) ^b^ | <0.001 |
| Insulin | 6533 (38.9) | 194 (24.7)^b^ | 1912 (35.0) | 2198 (41.7)^b^ | 1769 (42.4) ^b^ | 460 (41.1) ^b^ | <0.001 |
| Transfusion | 6157 (36.6) | 276 (35.1) | 2035 (37.3) | 1987 (37.7) | 1463 (35.1) ^b^ | 396 (35.4) | 0.10 |
| TPN | 1059 (6.3) | 59 (7.5) | 346 (6.3) | 320 (6.1) | 259 (6.2) | 75 (6.7) | 0.61 |
| **Length of stay** |  |  |  |  |  |  |  |
| ICU LOS, median (Q1-Q3) | 2.3 (1.2-4.8) | 2.3 (1.1-4.4) | 2.3 (1.2-4.8) | 2.3 (1.2-4.7) | 2.2 (1.2-4.7) | 2.8 (1.4-5.5) ^b^ | 0.04 |
| Hospital LOS, median (Q1-Q3) | 8 (4-13) | 8 (4-14) | 8 (5-13) | 7 (4-13) | 7 (4-13) | 8 (5-14) | 0.86 |
| **Mortality^f^** |  |  |  |  |  |  |  |
| Hospital mortality, N (%) | 2047 (12.2) | 148 (18.8)^b^ | 799 (14.6) | 551 (10.4)^b^ | 417 (10.0) ^b^ | 132 (11.8) ^b^ | <0.001 |
| Mortality 30 days, N (%) | 2339 (13.9) | 185 (23.5)^b^ | 910 (16.7) | 644 (12.2)^b^ | 447 (10.7) ^b^ | 153 (13.7) ^b^ | <0.001 |
| Mortality one year, N (%) | 4392 (26.1) | 363 (46.2)^b^ | 1720 (31.5) | 1203 (22.8)^b^ | 844 (20.3) ^b^ | 262 (23.4) ^b^ | <0.001 |
| ^a^p-value for association or trend across BMI categories; ^b^p<0.05 compared to normal weight;  ^c^ SAPS was calculated without the age component because age was an independent variable in the analysis; ^d^if smoker status was unknown, they were treated as "no." However, trends were the same when looking at those with data (known status); ^e^Obesity-related cancers – breast, colon, uterine, esophageal, pancreatic, ovarian, and kidney; ^f^the mortality numbers are cumulative, i.e., the one-year mortality group includes the 30-day mortality group. Likewise, the 30-day mortality group includes most of the patients who died in the hospital; however, the few patients that died in the hospital after 30 days were not included in the 30-day mortality. | | | | | | | |

| **Table S3.** Univariate analysis of initial SAPS distribution and 30-day mortality by body mass index (BMI) category with the severely obese patients examined as a separate group. | | | | | | | | | | | | |
| --- | --- | --- | --- | --- | --- | --- | --- | --- | --- | --- | --- | --- |
|  | Total  (16812) | | **Underweight**  **(786)** | | **Normal**  **(5463)** | | **Overweight**  **(5276)** | | **Obese**  **(4168)** | | **Severely obese (1119)** | |
| **SAPS Category** (maximum possible = 56) | N (%) | Mortality % | N (%) | Mortality % | N (%) | Mortality % | N (%) | Mortality % | N (%) | Mortality % | N (%) | Mortality % |
| ≤4 | 1152 (6.9%) | 3.6% | 42  (5.3%) | 7.1% | 356  (6.5%) | 5.6% | 387  (7.3%) | 3.1% | 296 (7.1%) | 0.7%* | 71 (6.3%) | 5.6% |
| 5-6 | 1425  (8.5%) | 4.9% | 63  (8.0%) | 4.8% | 440  (8.1%) | 6.6% | 449  (8.5%) | 3.8% | 390 (9.4%) | 4.4% | 83 (7.4%) | 4.8% |
| 7-8 | 1969  (11.7%) | 7.4% | 93  (11.8%) | 19.4%* | 648  (11.9%) | 7.6% | 593  (11.2%) | 7.3% | 493 (11.8%) | 5.9% | 142 (12.7%) | 4.2% |
| 9-10 | 2193  (13.0%) | 9.7% | 117  (14.9%) | 14.5% | 731  (13.4%) | 12.7% | 694  (13.2%) | 9.2%* | 507 (12.2%) | 4.7%* | 144 (12.9%) | 9.7% |
| 11-12 | 2362  (14.0%) | 12.1% | 122  (15.5%) | 19.7% | 754  (13.8%) | 15.7% | 713  (13.5%) | 12.1%* | 614 (14.7%) | 7.3%* | 159 (14.2%) | 8.2%* |
| 13-14 | 2321  (13.8%) | 13.1% | 95  (12.1%) | 23.2% | 732  (13.4%) | 16.3% | 740  (14.0%) | 11.0%* | 600 (14.4%) | 10.8%* | 154 (13.8%) | 10.4% |
| 15-16 | 2016  (12.0%) | 16.6% | 92  (11.7%) | 35.9%* | 629  (11.5%) | 19.9% | 672  (12.7%) | 14.7%* | 473 (11.4%) | 10.6%* | 150 (13.4%) | 18.7% |
| 17-18 | 1511  (9.0%) | 18.1% | 62  (7.9%) | 24.2% | 493  (9.0%) | 20.3% | 473  (9.0%) | 14.2%* | 381 (9.1%) | 18.1% | 102 (9.1%) | 22.6% |
| 19-20 | 862  (5.1%) | 23.1% | 47  (6.0%) | 40.4%* | 309  (5.7%) | 26.2% | 268  (5.1%) | 19.4% | 189 (4.5%) | 21.2% | 49 (4.4%) | 14.3% |
| >20 (21-35)** | 1001  (6.0%) | 47.4% | 53  (6.7%) | 58.5% | 371  (6.8%) | 47.4% | 287  (5.4%) | 42.9% | 225 (5.4%) | 47.1% | 65 (5.8%) | 58.5% |
| *p<0.05 compared to normal weight;**we followed the original SAPS study which grouped all of the patients with SAPS>20 in one category, and the maximum SAPS we observed was 35. | | | | | | | | | | | | |

| **Table S4**. Univariate analysis of the distribution of significant underlying diseases and 30-day mortality by body mass index (BMI) category with the severely obese patients examined as a separate group. | | | | | | | | | | | | |
| --- | --- | --- | --- | --- | --- | --- | --- | --- | --- | --- | --- | --- |
|  | Total  (16812) | | **Underweight**  **(786)** | | **Normal**  **(5463)** | | **Overweight**  **(5276)** | | **Obese**  **(4168)** | | **Severely obese (1119)** | |
| **Diagnosis**  **(ICD-9 code(s))** | N (%) | Mortality % | N (%) | Mortality % | N (%) | Mortality % | N (%) | Mortality % | N (%) | Mortality % | N (%) | Mortality % |
| HIV (042) | 195 (1.1%) | 14.9% | 36  (4.6%) | 8.3% | 102  (1.9%) | 13.7% | 42  (0.8%) | 23.8% | 11 (0.3%) | 9.1% | 4 (0.4%) | 25.0% |
| Leukemia/lymphoma/multiple myeloma (200-208) | 449 (2.7%) | 28.5% | 30  (3.8%) | 40.0% | 160  (2.9%) | 31.3% | 134  (2.5%) | 21.6% | 99 (2.4%) | 27.3% | 26 (2.3%) | 38.5% |
| Metastatic cancer (196-198) | 911 (5.4%) | 30.9% | 61  (7.8%) | 31.2% | 350  (6.6%) | 36.3% | 251  (4.8%) | 28.7% | 201 (4.8%) | 24.4%* | 48 (4.3%) | 29.2% |
| *p<0.05 compared to normal weight | | | | | | | | | | | | |
